# Factors Affecting Patient Satisfaction with Complete Dentures

**DOI:** 10.1155/2022/9565320

**Published:** 2022-04-08

**Authors:** Yara Oweis, Nadia Ereifej, Ayah Al-Asmar, Abdaljaber Nedal

**Affiliations:** Faculty of Dentistry, University of Jordan, Amman, Jordan

## Abstract

**Background:**

Rehabilitation of the edentulous patients has always been a challenge. The success of conventional complete denture therapy might be affected by several factors such as patients' age, personality, previous denture wearing experience, expectations, aesthetics, residual ridge form and anatomy, denture quality, the method of its construction, dentist experience, and dentist-patient relations.

**Objectives:**

The aim of this study was to compare patient satisfaction with complete dentures provided by fourth and fifth year dental students and prosthodontists with a minimum of 5 years' experience at the University of Jordan Hospital 8 weeks after insertion.

**Methods:**

Questionnaires were distributed to a total of 60 completely edentulous patients who received complete maxillary and mandibular dentures. Besides demographic questions, the questionnaire included questions that probed the patient's satisfaction with his maxillary and mandibular dentures in general using two types of scales.

**Results:**

Results indicated that dentist experience does not affect patients' satisfaction with their complete dentures. Our results also indicated that patients who had previous dentures could adapt more easily and were generally more satisfied with their newly inserted dentures especially with regard to their chewing ability and comfort with their mandibular dentures. Gender also influenced patient satisfaction with complete dentures especially the part related to psychological and social disability.

**Conclusions:**

Results of this study will help in further understanding factors influencing patient satisfaction with their complete dentures.

## 1. Background

Rehabilitation of the edentulous patients has always been a challenge. Edentulism, which has both functional and psychosocial consequences, can be corrected with the placement of removable dentures [1]. The success of this treatment modality might be affected not only by the patients' acceptance of his new dentures, but by his ability to use them which depends to a great extent on the quality of those dentures [2]. Accordingly, the success of conventional complete denture therapy might be affected by several factors such as patients' age, personality, previous denture wearing experience, expectations, aesthetics, residual ridge form and anatomy, denture quality, the method of its construction, dentist experience, and dentist-patient relations [3, 4].

Among the factors that might affect patients' acceptance of their new dentures is the dentists' experience. Several clinicians and patients believe that the success of dental treatments could be affected by the dentist experience; however, the results on this issue are inconclusive. While Evans et al. reported no significant correlation between the treatment outcomes and the experience of the surgeon [5], Gueders and Geerts found that the operator experience has a significant influence on microleakage in class V composite restorations [6]. Furthermore, the high-quality complete dentures provided by experienced dentists may not lead to patient satisfaction; therefore, it is difficult to evaluate the significance of experience in the field of denture treatment [4].

At the Faculty of Dentistry, University of Jordan, students start treating patients requiring complete dentures during their fourth year. During their third year, students acquire the required training in a preclinical setting where they practice on manikins the various steps of complete denture construction. In their fourth year, they do their first complete denture under the supervision of staff members who are qualified prosthodontists. Each student should deliver at least two complete dentures before the end of the fourth year. In their fifth year, students are required to do another two complete dentures and graduate with a minimum of four patients treated with complete dentures. During their clinical sessions, students are required to perform the different steps of complete denture construction on their own. The attending staff usually verifies if the procedure was done correctly and advices on any adjustments needed before the work is sent to the lab. At the end of each session, the student is graded for his performance during that session. After delivering the dentures to their patients, a follow-up session is usually scheduled one week later to adjust any issues related to complaints by the patients. Previous studies have reported that around 60% of experienced denture wearers are able to function satisfactorily within a week after their new dentures are fitted; however, 20% of these patients required up to 1 month to become proficient [2]. Accordingly, there exists lack of follow-up to these patients especially that a period of 6 to 8 weeks has been regarded as necessary to assess satisfactory use with the new dentures, as this period has the potential to establish new memory patterns for the masticatory muscles [2].

Success of treatment with complete dentures is often assessed differently by dentists and patients [7]. Sghaireen and Al-Omiri found that 10% of the subjects were not satisfied with their technically successful removable dentures [8]. Accordingly, clinical success of denture treatment can be assessed in terms of patient satisfaction. Satisfaction outcomes are easy to measure and allow direct quantification of patients' opinions and feelings towards different aspects of prosthodontic treatment. Satisfaction measures were found to be positively associated with oral health related quality of life (OHRQoL) [9–11]. The Oral Health Impact Profile (OHIP) is a questionnaire on oral health related quality of life which has been used as an effective means for comparing prosthodontic outcomes [1, 12]. The original (49-item) OHIP was developed by Locker and Slade. It included seven domains: functional limitation, physical pain, psychological discomfort, physical disability, psychological disability, social disability, and handicap [13]. In this study we use two types to of measures to probe patient satisfaction with their complete maxillary and mandibular dentures. A horizontal 100 mm visual analogue scale (VAS) translated into the Arabic language for ease of reading and understanding and a translated and modified OHIP-EDENT which consisted of 20 items adapted from the original 49-item OHIP-EDENT were used [14]. Our null hypothesis is that dentist experience does not affect patient satisfaction with their complete dentures and that patient previous experience is a decisive factor in patient satisfaction with their complete denture.

## 2. Methods

### 2.1. Study Population

The present study was reviewed and approved by the Deanship of Academic Research at the University of Jordan and the Jordan University Hospital (IRB number: 75/2019/2670) and registered at ClinicalTrials.gov (ID: https://clinicaltrials.gov/ct2/show/NCT05076968). All participants gave their informed consent prior to their inclusion in the study. The study population was completely edentulous patients attending the undergraduate students' clinic and specialty prosthodontic clinics at the Jordan University Hospital in need of conventional complete dentures. Inclusion criteria were patients aged 45–80 years seeking new conventional complete dentures for first time or as replacement of their previous complete dentures; patients who had been completely edentulous for a minimum of three months; patients without severe underlying medical conditions, neuromuscular dysfunction, auditory problems, mental conditions, oral pathology, xerostomia, or tied tongue condition. Each participant received new complete maxillary and mandibular dentures and was followed up for 1 week to make any necessary adjustments. The complete dentures were fabricated either by prosthodontists with a minimum of 5 years' experience (group 1, *n* = 30) or by undergraduate students under supervision (group 2, *n* = 30). After 8 weeks, the patients were reviewed and were given the questionnaire by a trained dentist, who did not participate in providing the treatment.

### 2.2. Questionnaire

The questionnaire consisted of 2 parts. The first part included demographic and contact information questions for the patient. The second part included questions that probed the patient's satisfaction with his maxillary and mandibular dentures in general which was also divided into two parts. In the first part, participants rated their general satisfaction with their denture on a horizontal 100 mm VAS in which 1 meant completely dissatisfied and 10 meant completely satisfied. The participants were asked to put a circle on the part of the scale that best represented their response. In the second part, participants filled out an Arabic translation of the OHIP-EDENT questionnaire. The OHIP-EDENT consisted of 20 items, which included functional limitation, physical pain, psychological discomfort, physical disability, psychological disability, social disability, and handicap. Each item was scored on a 1 to 5 scale in which 1 = “never,” 2 = “rarely,” 3 = “occasional,” 4 = “most of the time,” and 5 = “all the time.” The sum of the scores was computed, yielding a total OHIP ranging from 20 to 100, in which 20 represented the best possible score and 100 represented the worst possible score. At the end of the questionnaire some space was left for patients to express their experience with their dentures.

### 2.3. Statistical Analysis

Statistical analyses were conducted by using SPSS statistics software (IBM SPSS Statistics, v22.0; IBM Corp). Descriptive statistics were generated, and the data were inspected for normality using Shapiro–Wilk test. Since all the data did not follow a normal distribution, nonparametric Mann–Whitney *U* test was used at a significant level of *p* < 0.05. Pearson's rho test was used to examine correlations between groups. To label the strength of the association, for absolute values of rho, 0–0.19 was regarded as very weak, 0.2–0.39 as weak, 0.40–0.59 as moderate, 0.6–0.79 as strong, and 0.8–1 as very strong correlation.

## 3. Results

**Figure 1:**
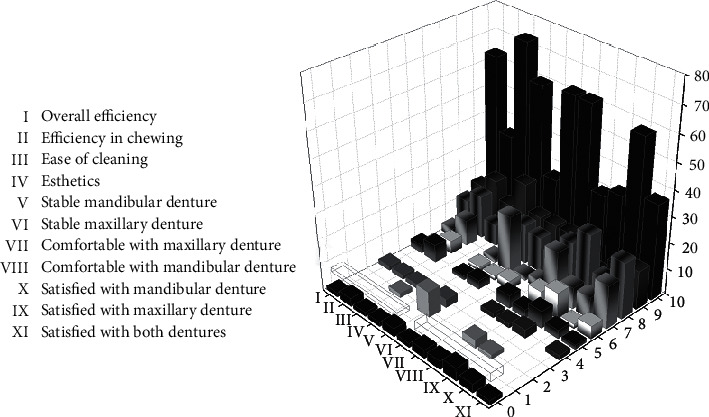
Percent distribution of the frequencies of the variables assessed by the patients in the 100 mm VAS scale.

**Figure 2:**
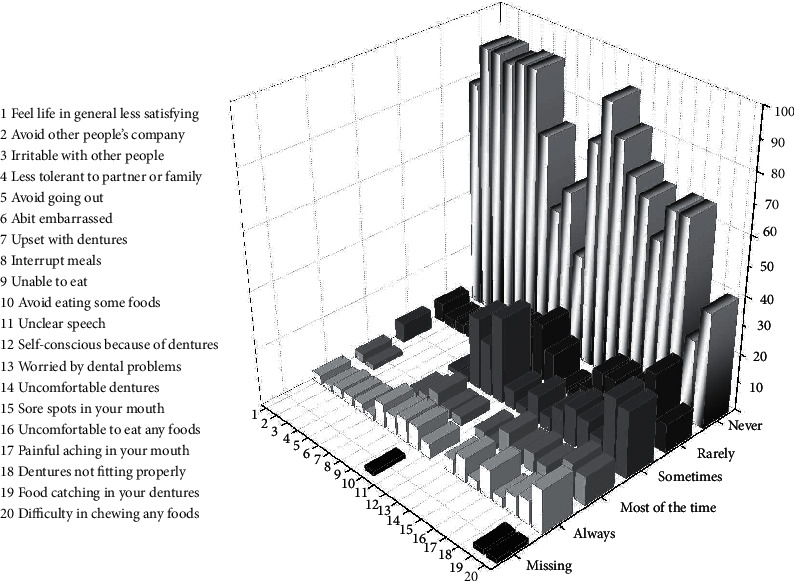
Percent distribution of the frequencies, for the variables assessed by the patients using the OHIP-EDENT scale.

The study sample consisted of 43 males (71.7%) and 17 females (28.3%) with a total of 60 patients, 50% of which received their treatment by a prosthodontist and the remaining 50% received their treatment by undergraduate 4^th^ and 5^th^ year students under the supervision of a prosthodontist. 60% of these patients were first-time denture wearers and 40% had previous dentures. Percent distributions of the frequencies, for the variables assessed by the patients (the 100 mm VAS and OHIP-EDENT), are shown in Figures 1 and 2 successively. The ratings of patient assessments of their dentures were generally high. More than half of the examined patients claimed all the examined variables to be in the highest category for both parts of the questionnaire. The parameters with the best ratings were ease of cleaning and overall satisfaction (Figure 1), being self-conscious, a bit embarrassed, avoid going out, irritable with other people, and avoid other people's company (Figure 2).

All correlations with dentist, previous experience, and gender were regarded as weak to very weak except the correlation between being satisfied with the mandibular denture and the dentist which showed a negative moderate correlation and that between gender and being satisfied with both dentures which showed a strong negative correlation. Statistically significant positive weak correlation (Pearson's correlation coefficient: 0.25, *p*=0.05) was found between the overall efficacy score and the previous experience with dentures. The other 100 mm VAS variables considered showed no significant correlation with dentist category, gender, or the previous experience with dentures (Table 1).

Statistically significant negative correlation (Pearson's correlation coefficient: −0.30, *p*=0.02) was found between the score of the difficulty in food-chewing and the previous experience with dentures. Statistically significant positive correlation was found between five of the variables considered (i.e., having sore spots in the mouth, being self-conscious because of dentures, avoidance of going out, being less tolerant to partner or family, being irritable with other people, and avoidance of other people's company) and the type of gender. The other OHIP-EDENT variables considered showed weak to very weak nonsignificant correlation with dentist category, gender, or the previous experience with dentures (Table 2).

Results of the median and Mann–Whitney *U* test for 100 mm VAS and OHIP-EDENT parts of the questionnaire are shown in Tables 3 and 4 successively. Results of 100 mm VAS showed a statistically significant difference in patient satisfaction with regard to mandibular denture stability, comfort, and efficiency in chewing when the patient had a previous denture. There was also a statistically significant difference in the denture overall efficiency between students and specialists' patients with students' patients scoring higher on the 100 mm VAS (Table 3). As for the OHIP-EDENT part of the questionnaire, there was a statistically significant difference in difficulty in chewing and total OHIP score with regard to previous experience. Moreover, a statistically significant difference was present between males and females for self-conscious about your teeth, a bit embarrassed, avoid going out, less tolerant to family, irritable with other people, and avoid other people's company.

Nine patients added their comments about their dentures at the end of the questionnaire. Two of the patients complained about food being impacted under the mandibular denture. One of the patients reported that he could not use his mandibular denture. 3 of the patients complained about mandibular denture being uncomfortable, one of the patients said that he could not use his dentures due to gag reflex, and one commented that he did not use his dentures without giving further explanation.

## 4. Discussion

Rehabilitation of edentulous patients with complete dentures has always been a great challenge to dentists. Satisfaction with complete dentures has been associated with several different denture-related, patient-related, and oral-related factors. Among these factors are general health, aesthetics, phonetics, experiences with previous dentures, and patient expectation regarding treatment which were evaluated in previous studies [15]. In this study, we investigated whether patient satisfaction is affected by the dentists' experience, previous patient experience, or gender 8 weeks after insertion. Results of this study confirmed our hypothesis that dentist experience does not affect patients' satisfaction with their complete dentures since there was no significant correlation between the dentists' experience and patients' satisfaction with their complete dentures. Our results also indicated that patients who had previous dentures could adapt more easily and were generally more satisfied with their newly inserted dentures especially with regard to their chewing ability and comfort with their mandibular dentures as clearly demonstrated through the significant positive correlation between the overall efficacy score and previous denture experience. Moreover, there was significant negative correlation between the score of the difficulty in food-chewing and the previous experience with dentures. Gender also had an effect on patient satisfaction with complete dentures especially the part related to psychological and social disability which was evident in the correlation results between five of the variables considered (i.e., having sore spots in the mouth, being self-conscious because of dentures, avoidance of going out, being less tolerant to partner or family, being irritable with other people, and avoidance of other people's company) and the type of gender.

In this study we used two types of scales to assess patient satisfaction with their complete dentures: the 100 mm VAS and the OHIP-EDENT. Previous studies indicated that the OHIP is a reliable and valid instrument suitable for assessment of OHRQoL and that the modified short version for edentulous patients, OHIP-EDENT, has measurement properties comparable with those of the OHIP-49 and is the most appropriate for edentulous patients [7, 16].

The distribution curve showing the patients' assessments related to their complete dentures is visibly skewed toward the highest score area as evident in Figures 1 and 2. This means that this treatment is quite successful in providing patients with acceptable dentures. This is in accordance with other studies which reported that the great majority of patients were satisfied with their complete dentures [17].

Previous studies have revealed that patient satisfaction is unrelated to denture quality and to different complete denture-making techniques [15]. Edentulous patients generally do not take into account their own baseline situation and expect new complete dentures to fit and function equally to, or even better than, their original natural teeth, in spite of the resorbed ridges, collapsed muscles, and other physical changes that had occurred in their oral cavities. New dentures provide an adequate solution to the patient's situation from the dentist's point of view; however, besides the patients' own baseline situation, the insertion of new dentures greatly impacts the patients' stomatognathic system [18]. Foreign object sensation, nausea, phonetic problems, and inability or difficulty in chewing and swallowing and excessive salivary flow are the common complaints of edentulous patients during the first few days after insertion of their complete dentures. As the complete dentures do not generally match the patients' expectations, the patients might no longer be willing to wear them.

In our study, patient satisfaction with their complete dentures was probed 8 weeks after insertion. Previous studies showed that although approximately 60% of experienced denture wearers were able to eat and speak satisfactorily within a week after replacement dentures were fitted, another 20% of these patients required up to 1 month to become proficient [19]. Accordingly, a period of 6 to 8 weeks has been regarded as necessary to assess satisfactory use with the new dentures, as this period has the potential to establish new memory patterns for the masticatory muscles [2].

The stability and retention of a denture are mainly affected by the base form of a denture, which depends on the impression taken by the dentist [4]. Patients often express dissatisfaction with their mandibular dentures. Complaints include decreased retention, stability, difficulty with mastication, and verbal communication [16]. This was in accordance with our results which indicated that patients were less satisfied with their mandibular dentures (Table 3). Moreover, many patients reported that they were not comfortable or less comfortable with their mandibular dentures and that food was impacted under their mandibular dentures and a few said that they could not use their mandibular denture. This is due to the smaller mandibular denture bearing area which is accompanied by resorption of bone in the mandibular arch as well as the difficulty in muscle control of the mandibular denture due to the presence of the tongue. For this reason, there is an increasing trend in providing patients with implant retained overdentures to enhance denture stability, retention, and masticatory efficiency [3]. Another less expensive solution the implant retained overdentures could be the use of CAD/CAM technology in the construction of the complete denture which includes two patient appointments. In the first, the required clinical data for denture construction are registered and in the second the dentures are inserted. Compared with standard denture construction CAD/CAM dentures involve simpler laboratory process, fewer dental appointments, and better clinical outcomes [20]. Studies have revealed that psychogenic factors, such as a good relationship between the patient and his dentist as well as patients' expectations of the outcome of the treatment, could be more important than anatomic, clinical, and technical factors that determine patient satisfaction with treatment [7]. This may be further understood on the basis of Carlsson's suggestion that patient-centred outcome scores are affected by not only dentists' technical skills but also patient-related psychological and emotional factors [21]. This was reflected in our results which indicated that patients treated by students found their dentures more efficient than those treated by specialists (Table 1). Patients treated by specialists might have had higher expectations of the treatment outcome which has affected their overall satisfaction with their dentures. Moreover, students tend to be more passionate and spend a good amount of time talking and listening to their patient and are really grateful and happy at the end of the day that these patients accepted to give them the chance to treat them which might have been reflected on patients.

## 5. Conclusions

Results of this study indicated that although adapting to new dentures is highly variable, previous patient experience and gender might remain the decisive factors with their dentures more than dentist experience. Occasionally, the high-quality complete dentures provided to a skilled prosthodontist might not lead to patient satisfaction; therefore, it is difficult to evaluate the significance of experience in the field of denture treatment.

### 5.1. Limitations

Several students and prosthodontists were involved in this study, which could have affected the results. The students are limited by the number of dentures they are required to deliver during the course of their study, which made it difficult for only one student to treat all 30 patients included in this study. Being also limited by the number of sessions, only one follow-up was done, which might have affected the patients' comfort. Furthermore, our sample was only taken from patients attending the university clinics and did not include other centers such as the public health sector or the private sector. Future studies might need to be conducted to include other sectors and more follow-ups.

## Figures and Tables

**Table 1:** Results of Pearson correlations along with *p* value measured at a significant level of *p* < 0.05 for dentist, gender, and previous experience versus all variables of the 100 mm VAS scale part of the questionnaire.

100 mm VAS	Dentist		Gender		Previous experience	
	Pearson	*p* value	Pearson	*p* value	Pearson	*p* value
Satisfied with both dentures	−0.19	0.80	−0.86	0.52	0.10	0.46
Satisfied with maxillary denture	−0.01	0.95	−0.07	0.60	−0.03	0.84
Satisfied with mandibular denture	−0.45	0.73	0.04	0.79	0.21	0.10
Comfortable with mandibular denture	0.05	0.70	−0.13	0.34	0.28	0.28
Comfortable with maxillary denture	0.02	0.91	−0.01	0.92	−0.04	0.74
Stable maxillary denture	−0.06	0.66	0.13	0.32	0.22	0.09
Stable mandibular denture	0.05	0.72	0–−0.10	0.48	0.24	0.07
Esthetics	−0.14	0.29	0.03	0.81	0.02	0.80
Ease of cleaning	0.06	0.64	−0.04	0.78	0.09	0.47
Efficiency in chewing	0.02	0.89	−0.20	0.13	0.22	0.09
Overall efficiency	0.15	0.27	−0.12	0.35	0.25	**0.05**

**Table 2:** Results of Pearson correlations along with *p* value measured at a significant level of *p* < 0.05 for dentist, gender, and previous experience versus all variables of the OHIP-EDENT part of the questionnaire.

OHIP-EDENT item	Dentist		Gender		Previous experience	
	Pearson	*p* value	Pearson	*p* value	Pearson	*p* value
Difficulty in chewing any foods	−0.12	0.35	0.06	0.7	−0.30	**0.02**
Food catching in your dentures	0.01	0.92	−0.002	0.99	−0.08	0.55
Dentures not fitting properly	0.16	0.22	0.16	0.22	−0.11	0.41
Painful aching in your mouth	−0.20	0.12	0.24	0.06	−0.11	0.39
Uncomfortable to eat any foods	0.06	0.64	0.07	0.56	−0.16	0.22
Sore spots in your mouth	−0.16	0.23	0.25	**0.05**	−0.13	0.31
Uncomfortable dentures	0.19	0.15	0.09	0.49	−0.20	0.12
Worried by dental problems	0.03	0.81	0.24	0.06	−0.13	0.31
Self-conscious because of dentures	0.03	0.84	0.40	**0.002**	−0.21	0.88
Unclear speech	−0.15	0.25	0.13	0.34	−0.13	0.32
Avoid eating some foods	−0.07	0.61	0.22	0.09	−0.22	0.09
Unable to eat	−0.05	0.69	0.09	0.49	−0.25	**0.05**
Interrupt meals	0.13	0.31	−0.05	0.71	−0.17	0.19
Upset with dentures	−0.04	0.79	0.19	0.14	−0.24	0.06
A bit embarrassed	−0.12	0.36	0.26	0.05	−0.12	0.37
Avoid going out	−0.004	0.98	0.31	**0.02**	−0.14	0.30
Less tolerant to partner or family	0.02	0.89	0.34	**0.01**	−0.11	0.42
Irritable with other people	−0.004	0.98	0.31	**0.02**	−0.14	0.30
Avoid other people's company	−0.03	0.82	0.31	**0.02**	−0.13	0.32
Feel life in general less satisfying	0.20	0.14	0.13	0.32	−0.04	0.77
Total OHIP	−0.001	0.9	0.24	0.07	−0.22	0.08

Bold indicates a statistically significant difference between groups.

**Table 3:** Results of the median and statistical analysis for the different questions included in the 100 mm VAS part of the questionnaire for the factors dentist, previous experience, and gender.

100 mm VAS item	Dentist			Previous experience			Gender		
	Sp	St	*p* value	Yes	No	*p* value	M	F	*p* value
Satisfied with both dentures	8.5	8.5	0.88	8	9	0.45	8.5	8.5	0.54
Satisfied with maxillary denture	9	10	0.49	10	10	0.98	10	10	0.86
Satisfied with mandibular denture	8	8	0.91	8	9	**0.05**	8	9.5	0.37
Comfortable with mandibular denture	8	8	0.59	7	9	**0.01**	8	8	0.44
Comfortable with maxillary denture	9.5	10	0.42	10	10	0.97	10	10	0.53
Stable maxillary denture	10	10	0.56	10	10	0.06	10	10	0.74
Stable mandibular denture	8	8	0.89	7	9	**0.01**	8	7.5	0.59
Esthetics	10	10	0.85	10	10	0.58	10	10	0.45
Ease of cleaning	10	10	0.31	10	10	0.20	10	10	0.71
Efficiency in chewing	8.5	9	0.22	8	10	**0.05**	9	9	0.35
Overall efficiency	9	10	**0.04**	10	10	0.14	10	10	0.77

Data were analyzed using Mann–Whitney *U* test at a significant level *p* < 0.05. Bold indicates statistically significant differences between groups. SP: specialist, St: student, M: male, and F: female.

**Table 4:** Results of the median and statistical analysis for the different questions included in the OHIP-EDENT part of the questionnaire for the factors dentist, previous experience, and gender.

OHIP-EDENT item	Dentist			Previous experience			Gender		
	Sp	St	*p* value	Yes	No	*p* value	M	F	*p* value
Difficulty in chewing any foods	3	1.5	0.28	3	1	**0.03**	2.5	2	0.72
Food catching in your dentures	2	2.5	0.94	2	2	0.73	2.5	2	0.79
Dentures not fitting properly	1	1	0.13	1	1	0.80	1	1	0.54
Painful aching in your mouth	1	1	0.09	1	1	0.51	1	1	0.20
Uncomfortable to eat any foods	1	1.5	0.55	1	1	0.42	1	1.5	0.67
Sore spots in your mouth	1	1	0.24	1	1	0.30	1	1.5	0.19
Uncomfortable dentures	1	1	0.14	1	1	0.36	1	1	0.45
Worried by dental problems	1	1	0.77	1	1	0.63	1	1	0.12
Self-conscious because of dentures	1	1	1.00	1	1	0.73	1	1	**0.01**
Unclear speech	1	1	0.21	1	1	0.62	1	1	0.82
Avoid eating some foods	2	2	0.35	3	2	0.07	2	2	0.09
Unable to eat	2	1	0.39	2	1	0.09	1	1.5	0.62
Interrupt meals	1	2	0.30	2	2	0.33	2	1	0.42
Upset with dentures	1	1	0.31	1	1	0.09	1	1	0.46
A bit embarrassed	1	1	0.61	1	1	0.92	1	1	**0.01**
Avoid going out	1	1	0.56	1	1	0.80	1	1	**0.03**
Less tolerant to partner or family	1	1	0.97	1	1	0.69	1	1	**0.01**
Irritable with other people	1	1	0.98	1	1	0.80	1	1	**0.03**
Avoid other people's company	1	1	0.54	1	1	0.80	1	1	**0.03**
Feel life in general less satisfying	1	1	0.17	1	1	0.90	1	1	0.28
Total OHIP	29.5	27.5	0.98	31	26	**0.04**	30	27.5	0.78

Data analyzed using Mann–Whitney *U* test at a significant level *p* < 0.05. Bold indicates statistically significant differences between groups. SP: specialist, St: student, M: male, and F: female.

## Data Availability

Data will be available from the corresponding author upon request.
